# Adverse Events After Surgical Treatment of Adult Diaphyseal Forearm Fractures: a Retrospective Analysis of 470 Patients

**DOI:** 10.2106/JBJS.OA.22.00115

**Published:** 2023-08-16

**Authors:** Henri Vasara, Samuli Aspinen, Jussi Kosola, Juha Sartanen, Tuomo Naalisvaara, Jan Myllykoski, Antti Stenroos

**Affiliations:** 1 Department of Hand Surgery, Helsinki University Hospital, University of Helsinki, Helsinki, Finland; 2 Department of Orthopedics and Traumatology, Kanta-Häme Central Hospital, Hämeenlinna, Finland; 3 Department of Orthopedics and Traumatology, South Karelia Central Hospital, Lappeenranta, Finland; 4 Department of Orthopedics and Traumatology, Helsinki University Hospital, University of Helsinki, Helsinki, Finland; 5 Department of Orthopedics and Traumatology, Päijät-Häme Central Hospital, Lahti, Finland

## Abstract

**Background:**

The incidence of and risk factors for adverse events after internal fixation of diaphyseal forearm fractures have not been well defined in the current literature. The objective of this study was to estimate the incidence of adverse events after diaphyseal forearm fracture surgery in adults and explore potential risk factors for adverse events.

**Methods:**

We conducted a retrospective, multicenter, cohort study in which we evaluated all diaphyseal forearm fractures between 2009 and 2019 in patients presenting to 4 trauma hospitals in southern Finland. Patients <16 years of age and fracture-dislocations were excluded. There were 470 patients included in this study. Patient records were evaluated to identify and analyze adverse events.

**Results:**

There were 202 patients with both-bone fractures, 164 patients with isolated ulnar fractures, and 104 patients with isolated radial fractures. In total, 146 patients (31%) experienced an adverse event; 83 (18%) had major adverse events (persistent or requiring surgical intervention). The patients underwent procedures performed by 185 different surgeons. The median number of operations for a single surgeon was 2 (range, 1 to 12). The most common major adverse events were plate and screw-related issues (6%), nonunion (5%), persistent nerve injuries (4%), and refractures (4%). Higher body mass index, Gustilo-Anderson type-II open fractures, both-bone fractures, isolated radial fractures, and operations performed by junior residents were found to be risk factors for adverse events in the multivariable analysis.

**Conclusions:**

Adverse events after diaphyseal forearm fracture surgery are common. We recommend concentrating these operations in a limited team of surgeons and restricting inexperienced surgeons from operating on these fractures without supervision.

**Level of Evidence:**

Therapeutic Level IV. See Instructions for Authors for a complete description of levels of evidence.

Loss of forearm function substantially affects the quality of life^1^. Therefore, a diaphyseal forearm fracture endangers the function of the affected extremity. Compared with distal forearm fractures, the injury mechanism in diaphyseal fractures is more often high-energy trauma, and diaphyseal fractures are more often seen in the working-age population^2,3^.

Consequently, adequate fracture reduction and fixation are crucial, and nonoperative treatment is rarely favored^4,5^. Indications for cast immobilization include isolated single-bone fractures with minimal displacement, angulation, and rotation and fractures that are considered inoperable because of considerable patient comorbidities^6^. Even minimally displaced both-bone forearm fractures are prone to more displacement, malunion, and nonunion^7^. Thus, open reduction and internal fixation with compression plating is used to manage most forearm fractures to enable rapid union and restoration of forearm anatomy^6^.

The long-term outcomes are excellent or satisfactory with properly assigned operative or nonoperative treatment, even in both-bone fractures^8^. However, operative treatment carries a risk of adverse events. The common adverse events include nonunion, surgical site infection, nerve injuries, and radioulnar synostosis^9-14^.

To our knowledge, no risk factors for adverse events have been identified, as all publications have had insufficient patient numbers for risk factor analysis. Despite the importance of forearm function in daily activities^15^, current knowledge regarding complications in these fractures is mainly premised on older publications^9-11^. Only a few articles have been published in the last decade^13,14,16^.

The objective of this study was to estimate the incidence of adverse events in surgically treated diaphyseal forearm fractures (both-bone, isolated radial, and isolated ulnar fractures) in adults and identify possible risk factors for adverse events.

## Materials and Methods

### Setting and Inclusion Criteria

**Fig. 1:**
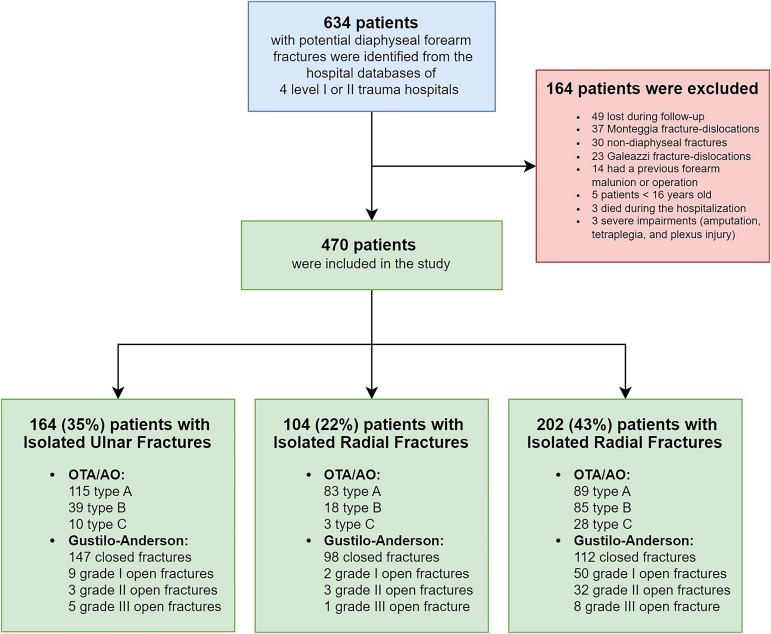
Patient inclusion flowchart.

We performed a retrospective, multicenter, cohort study of patients from 4 hospitals in southern Finland (1 Level-I trauma center and 3 Level-II trauma centers). The chosen hospitals are the only public hospitals in their area with an on-call orthopaedic surgeon at all times, with a population base of 2.2 million residents^17^. Between January 2009 and December 2019, patients who were ≥16 years of age and had diaphyseal forearm fractures were identified (see Appendix Table 1). The diaphyseal region of the forearm was defined on the basis of OTA/AO guidelines^18^. Furthermore, we excluded Galeazzi and Monteggia fracture-dislocations from the study. Comprehensive inclusion and exclusion criteria are presented in Figure 1. A total of 470 patients were included in the analysis.

The participating hospitals’ institutional boards approved this study. An ethical committee approval or informed patient consent was not required, as the study did not involve any patient interaction.

### Follow-up

The patient records were evaluated for a minimum of 2 years postoperatively. By protocol, only a single follow-up visit was performed routinely, and further follow-up visits were scheduled if needed. After the last follow-up visit, the patients were instructed to contact their physician should problems arise. The median length of clinical follow-up was 12.3 weeks (range, 21 days to 8.8 years), and the median number of follow-up visits was 2 (range, 1 to 15).

### Perioperative Information

We collected demographic data and comorbidities from the patient database (Table I). Standard anteroposterior and lateral forearm radiographs were available for all patients. In addition, computed tomography (CT) scans were examined if available. The authors classified all fractures according to the OTA/AO classification^18^. Open fractures were further classified on the basis of the Gustilo-Anderson classification system^19^ (Fig. 1).

**TABLE I:** Patient Characteristics*

	Patients with Isolated Ulnar Fractures (N = 164)	Patients with Isolated Radial Fractures (N = 104)	Patients with Both-Bone Fractures (N = 202)	Total (N = 470)
Patient demographic characteristics				
Age† *(yr)*	46 (33 to 57)	36 (27 to 50)	35 (24 to 51)	38 (27 to 54)
Male sex‡	106 (65%)	69 (66%)	127 (63%)	302 (64%)
Delay to surgery† *(days)*	4 (0 to 54)	1 (0 to 22)	1 (0 to 58)	2 (0 to 58)
BMI†,§ *(kg/m*^*2*^*)*	25 (17 to 59)	25 (16 to 32)	25 (16 to 40)	25 (16 to 59)
ASA class‡				
I	86 (52%)	71 (68%)	97 (48%)	254 (54%)
II	53 (32%)	24 (23%)	77 (38%)	154 (33%)
III	24 (15%)	9 (9%)	25 (12%)	58 (12%)
IV	1 (<1%)	0 (0%)	3 (1%)	4 (1%)
Fracture energy‡#				
Low	57 (36%)	40 (39%)	66 (33%)	163 (35%)
Moderate	68 (43%)	51 (49%)	90 (45%)	209 (45%)
High	35 (22%)	13 (13%)	44 (22%)	92 (20%)
Injury mechanism‡				
Falling <1 m	52 (32%)	39 (38%)	65 (32%)	156 (33%)
Falling 1 to 3 m	11 (7%)	7 (7%)	17 (8%)	35 (7%)
Falling >3 m	3 (2%)	1 (1%)	8 (4%)	12 (3%)
Car accident	13 (8%)	5 (5%)	18 (9%)	36 (8%)
Motorbike accident	10 (6%)	13 (13%)	20 (10%)	43 (9%)
Car hitting pedestrian or cyclist	5 (3%)	0 (0%)	3 (2%)	8 (2%)
Bicycle injury	11 (7%)	5 (5%)	24 (12%)	40 (9%)
Skateboard injury	1 (<1%)	2 (2%)	9 (5%)	12 (3%)
Crush injury	2 (1%)	7 (7%)	11 (5%)	20 (4%)
Torsion injury	4 (2%)	4 (4%)	3 (2%)	11 (2%)
Direct impact or assault	40 (24%)	10 (10%)	4 (2%)	54 (12%)
Contact sport injury	1 (<1%)	9 (9%)	10 (5%)	20 (4%)
Other or unspecified injury	11 (7%)	2 (2%)	10 (5%)	23 (5%)
Comorbidities‡				
Present smoking	30 (18%)	11 (11%)	27 (13%)	68 (15%)
Alcohol abuse	17 (10%)	5 (5%)	17 (8%)	39 (8%)
Intravenous drug abuse	7 (4%)	5 (5%)	8 (4%)	20 (4%)
Diabetes	13 (8%)	4 (4%)	7 (4%)	24 (5%)
Previous neuropathy	3 (2%)	0 (0%)	1 (<1%)	4 (1%)
Atherosclerosis	3 (2%)	0 (0%)	2 (1%)	5 (1%)
Other considerable injuries	41 (25%)	19 (18%)	61 (30%)	121 (26%)
Ipsilateral extremity injury	2 (1%)	12 (12%)	22 (11%)	36 (8%)
Primary implant used‡				
LCP	142 (87%)	77 (74%)	157 (78%)	376 (80%)
LC-DCP	7 (4%)	7 (7%)	16 (8%)	30 (6%)
VLP	0 (0%)	8 (8%)	11 (5%)	19 (4%)
Nonconventional plates	15 (9%)	12 (12%)	18 (9%)	45 (10%)
Other treatment-related factors				
Postoperative cast treatment‡	63 (38%)	53 (51%)	68 (34%)	184 (39%)
Casting time† *(days)*	21 (14 to 56)	21 (14 to 35)	28 (14 to 56)	21 (7 to 56)
Tourniquet use‡**	99 (70%)	77 (79%)	141 (87%)	317 (79%)
Institution‡				
Level-I trauma center	103 (63%)	64 (62%)	158 (78%)	325 (69%)
Level-II trauma center	61 (37%)	40 (39%)	44 (22%)	145 (31%)

* ASA = American Society of Anesthesiologists (classification for physical status) and VLP = volar locking plate.

† The values are given as the median, with the IQR in parentheses.

‡ The values are given as the number of patients, with the percentage (with respect to the group in question) in parentheses.

§ 200 patients had missing data: 75 in the ulnar fracture group, 51 in the radial fracture group, and 74 in the both-bone fracture group.

# 6 patients had missing data: 4 in the ulnar fracture group and 2 in the both-bone fracture group. Low-energy fracture indicates fracture energy equivalent to a fall from a standing height; moderate-energy fracture indicates a fall from 1 to 3 m, a motor vehicle injury with speed <30 km/hr, or the equivalent; and high-energy fracture indicates a fall from >3 m, a motor vehicle injury with speed >30 km/hr, or the equivalent.

** 69 patients had missing data: 23 in the ulnar fracture group, 7 in the radial fracture group, and 39 in the both-bone fracture group.

### Fracture Treatment

The treatment followed the surgical guidelines proposed by the AO Foundation^6^. All fractures were definitively managed with open reduction and internal fixation with plates. Prophylactic antibiotics were administered to all patients during anesthesia induction. A cast was placed according to the operating surgeon’s preference. Fracture reduction was considered adequate if angulation was <10°, rotation was <10°, and cortical displacement was <2 mm.

We recorded the operating surgeons’ status and divided them accordingly: junior resident, senior orthopaedic resident, senior hand surgery resident, orthopaedic consultant, and hand surgery consultant. Junior residents had <3 years of surgical experience, whereas senior residents had ≥3 years of experience, especially in the particular specialty. In addition, we recorded whether a consultant surgeon was supervising or assisting with the procedure and determined the overall number of diaphyseal forearm fracture procedures performed during the research period for each surgeon.

The primary fixation methods used in the operations are presented in Table I. Dynamic compression, semitubular, one-third tubular, and reconstruction plates were classified as nonconventional plates. An external fixator device was used as a temporary fixation method in 5 patients.

### Adverse Event Definitions

An adverse event was defined as any deviation from the normal postoperative course^20^. We recorded all postoperative adverse events and divided them into major and minor categories. Persistent adverse events that did not show apparent signs of resolution during the clinical follow-up and adverse events requiring secondary operations were considered major adverse events. We did not include preoperative adverse events as they depended largely on the injury. In cases with uncertainty regarding whether the adverse event was preoperative or postoperative, the adverse events were included in the analysis.

We assessed fracture union on the basis of follow-up radiographs. Radiographs for the patients included in the study were available until there were clear clinical and radiographic signs of fracture healing. A nonunion was diagnosed if fracture union required a secondary operation or took >6 months. The fracture union was considered delayed if it took 3 to 6 months. Surgical site infections were divided into deep and superficial infections according to the U.S. Centers for Disease Control and Prevention criteria^21^. A nerve injury was diagnosed in cases in which the nerve status had been intact preoperatively, but a physician observed paresthesia or paralysis postoperatively. The nerve damage was considered transient if it resolved during the first 4 months of follow-up. Other recorded adverse events included plate and screw-related issues and refractures (refracture after plate removal or peri-implant fracture). All secondary operations were performed in the operating room.

### Statistical Analysis

We reported descriptive statistics using cross-tabulations. Nominal variables are presented as counts and percentages. For variables with missing values, the percentages of the patients with values were compared. The skewness of the continuous variables was assessed with histograms and quantile-quantile (Q-Q) plots. As all continuous variables were skewed, we present them as medians and interquartile ranges (IQRs). We used the binominal proportional interval to calculate the 95% confidence intervals (CIs) for adverse event proportions. Risk factor analysis was performed using binary logistic regression. Odds ratios (ORs) were calculated for each potential risk factor. Pairwise deletion was conducted in the case of missing values. We performed univariable modeling and selected potential variables to undergo multivariable logistic regression. To reduce bias in the covariate selection, we constructed a directed acyclic graph (see Appendix Figure 1). The covariate selection was performed utilizing DAGitty 3.0 software, developed by Textor et al.^22^, using a method proposed by Shrier and Platt^23^. A separate multivariable model was constructed for each tested variable. Furthermore, we reported the p values using the Wald test for nominal values and the Mann-Whitney U test for continuous variables. Significance was set at p < 0.05. Statistical analysis was conducted using SPSS version 27.0.1 (IBM).

### Source of Funding

No external funding was received for this study.

## Results

**Fig. 2:**
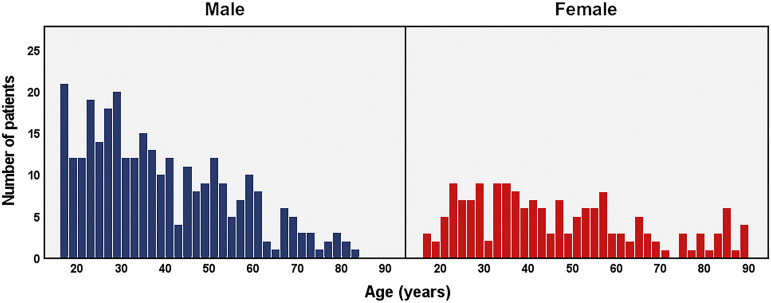
Age and sex distribution of surgically treated diaphyseal forearm fractures. Each bar represents 2 years.

There were 202 patients with both-bone fractures, 164 patients with isolated ulnar fractures, and 104 patients with isolated radial fractures. The median age of the patients was 38 years (IQR, 27 to 54 years), and 64% of the patients were male. The male patients were generally younger (36 years [IQR, 25 to 50 years]) than female patients (42 years [IQR, 30 to 58 years]) (p < 0.001) (Fig. 2).

The patients underwent operations performed by 185 different surgeons. The median number of operations for a single surgeon was 2 (IQR, 2 to 6 [range, 1 to 12]). Furthermore, orthopaedic consultants operated on 177 patients (38%); hand surgery consultants, on 25 (5%); senior orthopaedic residents, on 204 (43%); senior hand surgery residents, on 16 (3%); and junior residents, on 43 (9%); data were missing for the remaining 5 patients. In 39 (15%) of the 263 cases in which residents operated, a consultant supervised the whole operation. The anatomical reduction was considered adequate in 464 operations (99%).

### Adverse Events

A total of 146 patients (31% [95% CI, 27% to 35%]) encountered adverse events (Table II). In 66 patients, all adverse events were considered minor and resolved during the follow-up without further interventions, whereas 83 patients (18% [95% CI, 14% to 21%]) had major adverse events. Adverse events were more common in both-bone fractures (40% [95% CI, 33% to 47%]; p < 0.001) and isolated radial fractures (31% [95% CI, 23% to 40%]; p = 0.04) compared with isolated ulnar fractures (21% [95% CI, 15% to 27%]). Open fractures had an adverse event incidence of 43% (95% CI, 35% to 53%). Furthermore, 56 patients (12% [95% CI, 9% to 15%]) underwent at least 1 secondary operation due to an adverse event (Table III).

**TABLE II:** Adverse Events Categorized by the Fracture Site*

	Patients with Isolated Ulnar Fractures† (N = 164)	Patients with Isolated Radial Fractures† (N = 104)	Patients with Both-Bone Fractures† (N = 202)	P Value‡	Total† (N = 470)
No. of patients with adverse events	34 (21%)	32 (31%)	80 (40%)	**<0.001**	146 (31%)
No. of adverse events	37	40	111		188
Patients with major adverse events	19 (12%)	16 (15%)	48 (24%)	**0.009**	83 (18%)
Major adverse events					
Nonunion§	6 (4%)	3 (3%)	14 (7%)	0.20	23 (5%)
Persistent nerve injury	1 (<1%)	2 (2%)	14 (7%)	**0.002**	17 (4%)
Deep surgical site infection	2 (1%)	2 (2%)	6 (3%)	0.51	10 (2%)
Plate and screw-related issues	9 (5%)	5 (5%)	13 (6%)	0.85	27 (6%)
Fixation failure	3 (2%)	0 (0%)	8 (4%)		11 (2%)
Unsatisfactory reduction#	2 (1%)	3 (3%)	2 (1%)		7 (2%)
Unsatisfactory plate positioning#	3 (2%)	0 (0%)	0 (0%)		3 (<1%)
Irritation due to prominent screws	1 (<1%)	2 (2%)	3 (2%)		6 (1%)
Refracture	3 (2%)	3 (3%)	11 (5%)	0.17	17 (4%)
Peri-implant fracture	3 (2%)	2 (2%)	8 (4%)		13 (3%)
Refracture after plate removal	0 (0%)	1 (1%)	3 (2%)		4 (1%)
Non-plate-related rotational deficiency#	0 (0%)	2 (2%)	1 (<1%)	NA	3 (<1%)
Radioulnar synostosis#	1 (<1%)	0 (0%)	1 (<1%)	NA	2 (<1%)
Complex regional pain syndrome	0 (0%)	0 (0%)	2 (1%)	NA	2 (<1%)
Total	22	17	62		101
Patients with only minor adverse events	15 (9%)	18 (17%)	33 (16%)	0.08	66 (14%)
Minor adverse events					
Delayed union**	7 (4%)	10 (10%)	22 (11%)	0.06	39 (8%)
Transient nerve injury††	3 (2%)	10 (10%)	17 (8%)	**0.01**	30 (6%)
Superficial surgical site infection	5 (3%)	3 (3%)	10 (5%)	0.55	18 (4%)
Total	15	23	49		87

* NA = not applicable.

† The values are given as the number of patients or the number of adverse events, with or without the percentage in parentheses; a single patient might have multiple adverse events.

‡ Significant values are shown in bold.

§ Fracture nonunion required a secondary operation and clinical and radiographic nonunion took >6 months.

# These adverse events led to rotational deficiency.

** Fracture delayed union took >3 but <6 months to resolve.

†† These injuries resolved during the first 4 months of follow-up.

**TABLE III:** Secondary Operations Due to Adverse Events Categorized by Fracture Site

	Patients with Isolated Ulnar Fractures (N = 164)	Patients with Isolated Radial Fractures (N = 104)	Patients with Both-Bone Fractures (N = 202)	Total (N = 470)
No. of patients*	17 (10%)	13 (13%)	26 (13%)	56 (12%)
Adverse event-related secondary operations				
Refixation	9	7	16	32
Due to unsatisfactory reduction	2	2	1	5
Due to nonunion (treatment included bone graft)	3	3	7	13
Due to nonunion and fixation failure (treatment included bone graft)	3	0	5	8
Due to fixation failure	0	0	2	2
Due to refracture after plate removal	1	2	1	4
Revision due to infection	2	3	9	14
Peri-implant fracture surgery	2	2	7	11
Plate removal†	3	1	2	6
Screw removal	1	2	2	5
Corrective osteotomy	0	1	2	3
Other	1‡	1§	2#	4
Total	18	17	40	75

* The values are given as the number of patients, with the percentage in parentheses; a single patient might have multiple secondary operations.

† The reasons for plate removal were unsatisfactory placement (n = 3), unspecified pain (n = 2), and infection (n = 1).

‡ Radioulnar synostosis excision.

§ Anterior interosseus nerve suturing.

# A fibular graft to the radius after osteomyelitis and a fasciotomy.

Nonunion was diagnosed in 23 patients (5% [95% CI, 3% to 7%]), and delayed union (3 to 6 months) was seen in 39 patients. In total, 18 patients with nonunion required at least 1 secondary operation (refixation with a bone graft) to achieve union, 2 of whom required multiple reoperations. The 5 patients with nonoperatively treated nonunion, all of whom had both-bone fractures, achieved satisfactory fracture union eventually (range, 45 to 81 weeks). With regard to possible risk factors for nonunion, all patients had sufficiently anatomical reduction, although 4 were managed with nonconventional plates. In addition, 8 had sustained an open fracture, 2 had had a deep infection, 4 smoked cigarettes, and 4 had a history of alcohol abuse.

Surgical site infection was diagnosed in 28 patients (6% [95% CI, 4% to 9%]), of whom 10 had deep surgical site infections and 18 had superficial surgical site infections; 12 of these patients had had an open fracture. Nine patients developed a deep infection following the primary operation, and 1 patient developed a deep infection following a secondary surgical procedure after nonunion. The patients with deep infections underwent a median of 1.5 revision surgical procedures (range, 1 to 2 procedures) to treat the infection. One patient with a grade-I, open, both-bone fracture developed osteomyelitis to the radius and required plate removal and external fixator application. Subsequently, a reconstruction of the radius was performed with a fibular autograft. The other 9 patients were treated successfully without plate removal. Bacterial cultures were available in 6 deep infections; 2 of these were polymicrobial infections (see Appendix Table 2).

There were 47 patients (10% [95% CI, 8% to 13%]) with nerve injuries. Of these, 30 (6% [95% CI, 4% to 9%]) had transient injuries that resolved during the first 4 months of follow-up. Of the 17 persistent nerve injuries (4% [95% CI, 2% to 6%]), there were 5 affecting the median nerve, 5 affecting the radial nerve, 2 affecting the ulnar nerve, and 5 affecting multiple nerve branches (see Appendix Figure 2). Neurodiagnostic electroneuromyography was used to confirm the nerve damage in 15 cases. Of the persistent nerve injuries, 8 were considered sensory, 3 were considered motor, and 6 were considered combined. A nerve reconstruction surgeon was consulted in 10 cases, and end-to-end suturing was performed in 2 cases to treat iatrogenic nerve damage, 1 of which was performed during the initial operation. The other patients were managed nonoperatively.

There were 11 patients (2% [95% CI, 1% to 4%]) with fixation failure, of whom 8 were diagnosed as having a nonunion. All but 1 were managed with refixation (bone substitute was used with the 8 patients with nonunion). The remaining single fixation failure healed in a nonanatomical position with cast treatment. There were 15 patients whose main symptom was rotational deficiency. Seven of these patients had an unsatisfactory anatomical reduction and regained sufficient rotation after a secondary operation (5 early refixations and 2 corrective osteotomies), and 3 had unsatisfactory plate positioning and gained sufficient rotation after plate removal. Of the remaining patients with rotational deficiency, 2 had radioulnar synostosis, 2 were suspected of having soft-tissue scarring, and 1 had an unknown cause.

During their follow-up period, 17 patients (4% [95% CI, 2% to 6%]) sustained a new fracture (13 peri-implant fractures and 4 refractures after plate removal). In total, the plate was removed from 21 patients, of whom 6 had an adverse event and 15 had removal at the patient’s request with no compulsory clinical implications.

### Risk Factor Analysis

The results of the univariable analysis are presented in Table IV. In the multivariable analysis, higher body mass index (BMI), both-bone and isolated radial fractures (compared with isolated ulnar fractures), type-II open fractures, and operations performed by junior residents were found to be risk factors for adverse events. When compared with a locking compression plate (LCP), nonconventional plates showed a nonsignificant trend toward more adverse events, whereas a limited contact dynamic compression plate (LC-DCP) showed a nonsignificant trend toward fewer adverse events (Table V).

**TABLE IV:** Risk of Adverse Events and Univariable Binary Logistic Regression Analysis*

	Risk of Adverse Events†	OR‡	P Value§
Age in yr			
16 to 39	28% (72 of 254)	Reference	Reference
40 to 64	39% (62 of 159)	1.6 (1.1 to 2.5)	**0.03**
≥65	21% (12 of 57)	0.7 (0.3 to 1.3)	0.26
ASA class			
I	28% (71 of 253)	Reference	Reference
II	34% (52 of 153)	1.3 (0.9 to 2.0)	0.21
III	33% (19 of 58)	1.2 (0.7 to 2.3)	0.48
IV	50% (2 of 4)	2.6 (0.4 to 19)	0.35
Patient demographic characteristics			
Male sex	33% (99 of 302)	1.3 (0.8 to 1.9)	0.28
BMI (per 1-kg/m^2^ increase)		1.07 (1.02 to 1.12)	**0.01**
Obesity (BMI ≥30 kg/m^2^)	42% (20 of 48)	1.8 (0.9 to 3.4)	0.07
Diabetes	25% (6 of 24)	0.7 (0.3 to 1.9)	0.51
Alcohol abuse	31% (12 of 39)	1.0 (0.5 to 2.0)	0.99
Present smoking	35% (24 of 68)	1.2 (0.7 to 2.1)	0.42
Ipsilateral extremity injury	47% (17 of 36)	2.1 (1.1 to 4.2)	**0.03**
Fracture site			
Isolated ulna	21% (34 of 164)	Reference	Reference
Isolated radius	31% (32 of 104)	1.7 (1.0 to 3.0)	0.06
Both bones	40% (80 of 202)	2.5 (1.6 to 4.0)	**<0.001**
Fracture energy			
Low	25% (54 of 213)	Reference	Reference
Moderate	35% (55 of 159)	1.6 (1.0 to 2.4)	0.05
High	38% (34 of 89)	1.8 (1.1 to 3.1)	**0.03**
OTA/AO classification			
Type A	26% (75 of 287)	Reference	Reference
Type B	39% (56 of 142)	1.8 (1.2 to 2.8)	**0.005**
Type C	37% (15 of 41)	1.6 (0.8 to 3.2)	0.6
Fracture type			
Closed fracture	27% (97 of 357)	Reference	Reference
Open fracture			
Type I	39% (24 of 61)	1.7 (1.0 to 3.1)	0.06
Type II	50% (19 of 38)	2.7 (1.4 to 5.2)	**0.004**
Type III	43% (6 of 14)	2.0 (0.7 to 5.9)	0.20
Other treatment-related factors			
Tourniquet use	31% (97 of 317)	1.0 (0.6 to 1.8)	0.88
Cast treatment	31% (57 of 184)	1.0 (0.7 to 1.5)	0.97
Time from presentation to operation			
Emergency duty	35% (28 of 79)	Reference	Reference
Office hours, 0 to 2 days	35% (78 of 222)	1.0 (0.6 to 1.7)	0.96
Office hours, >2 days	24% (40 of 169)	0.6 (0.3 to 1.0)	0.05
Primary fixation implant			
LCP	30% (114 of 377)	Reference	Reference
LC-DCP	16% (5 of 31)	0.4 (0.2 to 1.2)	0.11
VLP	39% (10 of 26)	1.4 (0.6 to 3.3)	0.37
Nonconventional plates#	50% (17 of 34)	2.3 (1.1 to 4.7)	**0.02**
Hospital surgery volume			
Level-I trauma center	29% (95 of 335)	Reference	Reference
Level-II trauma center	35% (51 of 145)	1.4 (0.9 to 2.1)	0.12
Surgeon status**			
Orthopaedic consultant	29% (52 of 177)	Reference	Reference
Hand surgery consultant	28% (7 of 25)	0.9 (0.4 to 2.4)	0.88
Orthopaedic senior resident	30% (61 of 204)	1.0 (0.7 to 1.6)	0.91
Hand surgery senior resident	31% (5 of 16)	1.1 (0.4 to 3.3)	0.88
Junior resident	47% (20 of 43)	2.1 (1.2 to 4.1)	**0.03**

* ASA = American Society of Anesthesiologists and VLP = volar locking plate.

† The values are given as the risk of adverse events, with the number of adverse events divided by the number of patients predisposed to the adverse event in parentheses.

‡ The values are given as the OR, with the 95% CI in parentheses. In binominal variables, the non-predisposed group was used as a reference.

§ Significant values are shown in bold.

# Nonconventional plates include dynamic compression, one-third tubular, semitubular, and reconstruction plates.

** Junior residents have <3 years of surgical experience, and senior residents have ≥3 years of surgical experience. In Finland, one can specialize in hand surgery directly, with no previous specialist degree.

**TABLE V:** Multivariable Binary Logistic Regression for Adjusted ORs*

	Adjusted OR†	P Value‡
Patient demographic characteristics§		
BMI (per 1-kg/m^2^ increase)	1.07 (1.02 to 1.13)	**0.007**
Obesity (BMI ≥30 kg/m^2^)	1.8 (1.0 to 3.5)	0.07
Alcohol abuse	1.0 (0.5 to 2.0)	0.90
Present smoking	1.2 (0.7 to 2.1)	0.52
Fracture site#		
Isolated ulna	Reference	Reference
Isolated radius	2.6 (1.4 to 4.9)	**0.003**
Both bones	3.8 (2.1 to 6.7)	**<0.001**
Fracture type**		
Closed fracture	Reference	Reference
Open fracture		
Type I	1.6 (0.9 to 2.8)	0.13
Type II	2.4 (1.2 to 4.8)	**0.02**
Type III	1.8 (0.6 to 5.5)	0.31
Primary fixation implant††		
LCP	Reference	Reference
LC-DCP	0.4 (0.1 to 1.0)	0.06
VLP	1.3 (0.6 to 3.1)	0.54
Nonconventional plates‡‡	2.1 (1.0 to 4.4)	0.06
Surgeon status§§##		
Orthopaedic consultant	Reference	Reference
Hand surgery consultant	1.1 (0.4 to 2.8)	0.90
Orthopaedic senior resident	1.2 (0.7 to 2.0)	0.43
Hand surgery senior resident	1.4 (0.4 to 4.6)	0.59
Junior resident	4.4 (1.9 to 10.3)	**<0.001**
OTA/AO classification***	NA	NA

* VLP = volar locking plate and NA = not applicable.

† The values are given as the adjusted ORs, with the 95% CIs in parentheses.

‡ Significant values are shown in bold.

§ The covariates adjusted were age and sex.

# The covariates adjusted were fracture energy and injury mechanism.

** The covariates adjusted were fracture energy and OTA/AO classification.

†† The covariates adjusted were fracture site, OTA/AO classification, and injury energy.

‡‡ Nonconventional plates include dynamic compression, one-third tubular, semitubular, and reconstruction plates.

§§ Junior residents have <3 years of surgical experience, and senior residents have ≥3 years of surgical experience. In Finland, one can specialize in hand surgery directly, with no previous specialist degree.

## The covariates adjusted were fracture site, OTA/AO classification, number of operations per surgeon, and presence of consultant as a supervisor or an assistant.

*** The total effect could not be estimated using an adjustment, so the analysis was not performed.

## Discussion

Adverse events after surgically treated diaphyseal forearm fractures are common. In the present study, 18% of the patients experienced major adverse events, and 31% had some sort of negative deviation in their postoperative course. Furthermore, 12% required at least 1 secondary operation due to adverse events. Adverse events were more common in both-bone and open fractures, with adverse event incidences as high as 40% for both-bone fractures and 43% for open fractures. The most frequent major adverse events were plate and screw-related issues, nonunion, and nerve injuries.

In the limited number of publications since 2000, the combined adverse event incidence has been reported as 14% to 26% for diaphyseal forearm fractures using our criteria for adverse events^12,13,24,25^. However, the definitions of adverse events are often incomplete and vary between publications. Furthermore, those publications reported the incidence of adverse events separately, and minor adverse events and nerve injuries may have been underreported. Regardless, the adverse event incidence in the present study is high, even considering the factors affecting the comparison.

A concerning finding was that a single surgeon performed only a median of 2 operations during the study period. Even the surgeon with the most operations (12) would not qualify as a high-volume surgeon. A similar pattern was observed by Haas et al., as they had 108 different surgeons operating on 277 forearms^12^. It has been observed in several types of fractures that low-volume surgeons have a higher risk of adverse events, although evidence on forearm fractures has been lacking^26^. In our study, junior resident surgeons had significantly higher adverse event incidence than consultant surgeons (adjusted OR, 4.4 [95% CI, 1.9 to 10.3]), which indicates that patients who undergo procedures performed by inexperienced surgeons experience more adverse events. Based on our findings, we recommend concentrating these operations in a limited team of surgeons, restricting inexperienced surgeons from operating on these fractures without supervision, and performing complex cases with another surgeon.

Nerve injuries have been discussed rarely in the publications that reported on diaphyseal forearm fracture adverse events. To our knowledge, reliable nerve damage incidence has only been reported by Iacobellis and Biz^13^ and Marcheix et al.^14^. In their studies, nerve damage was present in 8% to 9% of the cases. However, the criteria for the nerve injuries were unclear, and Marcheix et al. did not report whether the nerve injuries resolved during the follow-up. The incidence of nerve injuries was greater in our study (10%), although most of these resolved spontaneously during the follow-up. Overall, nerve injuries appear to play a more substantial role in the adverse event burden than previously thought.

Nonunion has been recognized as a major adverse event of forearm fracture surgery since the first considerable publications^5,9^. Nonunion incidence has been reported as 2% to 9% in recent decades. Furthermore, an additional 4% to 9% of patients have been reported to have a delayed union^13,14,24,27^. The nonunion incidence in our institution has been highly comparable with the literature. Accordingly, nonunion and delayed union remain a considerable issue that has yet to be solved.

In our study, the incidence of surgical site infections was 6%, with a deep infection incidence of 2%. The incidence was especially high in patients with open fractures. In the literature, the overall incidence of surgical site infections has been 1% to 2%^10,12,14^, with corresponding open fracture incidences of 21% to 32%. Whereas treating superficial infections is routine, little has been published on deep infections. Marcheix et al. reported a single deep infection treated successfully with a revision surgical procedure^14^, and Haas et al. reported 3 deep infections; 1 was treated with plate removal, 1 was treated with a revision surgical procedure, and 1 was treated with only antibiotics^12^. Because the knowledge on treating these infections has been limited, general methods for surgical site infections must be adapted.

There were several limitations to the study. First, we acquired the data retrospectively from hospital databases, and patient-reported outcomes were not available. Therefore, defining the impact of adverse events in practice is not well defined. However, the adverse events were described with precision, and the incidences are accurate. Second, the mean length of active follow-up was brief, and the patients were not contacted afterward. Although some adverse events might have been treated in other hospitals or private clinics, the hospitals in the study are the primary trauma hospitals in their area and have a high re-referral percentage if an adverse event arises. Finally, as we conducted an exploratory study testing different risk factors, we had to perform multiple statistical tests. However, we took considerable measures to avoid bias in the statistical analysis.

In conclusion, adverse events after diaphyseal forearm fracture surgery, including major adverse events, are common (31% and 18%, respectively). We recommend concentrating these operations in a limited team of surgeons, restricting inexperienced surgeons from operating on these fractures without supervision, and performing the complex cases with another surgeon, especially in both-bone and open fractures.

## Appendix

Supporting material provided by the authors is posted with the online version of this article as a data supplement at jbjs.org (http://links.lww.com/JBJSOA/A550).
