# Erratum: Adverse Events After Surgical Treatment of Adult Diaphyseal Forearm Fractures. A Retrospective Analysis of 470 Patients

**DOI:** 10.2106/JBJS.OA.ER.22.00115

**Published:** 2024-04-05

*The Journal* publishes corrections when they are of significance to patient care, scientific data or record-keeping, or authorship, whether that error was made by an author, editor, or staff. Errata also appear in the online version and are attached to files downloaded from jbjs.org.

In the article entitled “Adverse Events After Surgical Treatment of Adult Diaphyseal Forearm Fractures. A Retrospective Analysis of 470 Patients” (JBJS OA. 2023;8[3]:e.22.00115), by Vasara et al., there was an error in Figure 1 of the article on page 2. In the bottom right of Figure 1, the text that had previously read “202 (43%) patients with Isolated Radial Fractures” should have read “202 (43%) patients with Both-Bone Fractures.”

